# Tackling Global Malnutrition and Hunger in the Final Push Toward the 2030 Agenda

**DOI:** 10.3390/nu17193059

**Published:** 2025-09-25

**Authors:** Stefania Moramarco, Ersilia Buonomo, Angela Andreoli, Leonardo Palombi

**Affiliations:** 1 Department of Biomedicine and Prevention, University of Rome “Tor Vergata”, 00133 Rome, Italyangela.andreoli@uniroma2.it (A.A.);; 2 Faculty of Medicine, Catholic University of “Our Lady of Good Counsel”, 1000 Tirana, Albania

**Keywords:** global nutrition targets, diet-related non-communicable disease targets, malnutrition, food insecurity, SDG 2-Zero Hunger, 2030 Agenda

## Abstract

Global malnutrition and hunger represent crises of alarming magnitude, threatening progress toward all the Sustainable Development Goals (SDGs). The drivers of food insecurity and malnutrition are complex and interconnected, including conflict, climate change, migration, population aging, and the erosion of social capital. Despite some progress in specific areas, current trends reveal insufficient advancement toward key global nutrition and diet-related, non-communicable disease targets, confirming the persistent double burden of malnutrition. Without urgent, multisectoral action—including investments in integrated nutrition policies, resilient food systems, and conflict resolution—the goal of achieving Zero Hunger by 2030 remains unlikely. The World Food Program estimates that in 2025, 319 million people will face acute food insecurity; if current trends persist, approximately 582 million people could still be chronically undernourished by 2030. Furthermore, overweight and obesity are projected to continue rising globally, with adult obesity prevalence expected to reach 19.8% in 2030. This narrative review synthesizes current global trends in malnutrition—both undernutrition and overnutrition—and food insecurity; it explores the root causes driving these crises and analyzes the scientific literature to inform future research in the critical years leading up to the 2030 Agenda deadline. It calls for coordinated global efforts that prioritize vulnerable populations, which are essential to reversing the current trajectory of malnutrition and hunger. Since nutrition is a fundamental component of sustainable development, achieving the SDG 2 targets is essential to the accomplishment of all 17 goals.

## 1. Introduction

Malnutrition encompasses conditions arising from deficits, excesses, or imbalances in an individual’s intake of energy and nutrients. It broadly includes three categories [1]:

- Undernutrition, comprising wasting (low weight-for-height), stunting (low height-for-age), and underweight (low weight-for-age). Undernutrition significantly increases children’s vulnerability to illness and mortality.
- Overnutrition, comprising overweight and obesity, resulting from an imbalance where energy intake exceeds energy expenditure. Overnutrition is a major risk factor for diet-related non-communicable diseases (NCDs), such as cardiovascular diseases, strokes, diabetes, and some cancers.
- Micronutrient-related malnutrition, encompassing both deficiencies (lack of vital vitamins and minerals) and excessive intake of certain micronutrients.

All forms of malnutrition share common underlying causes, making integrated interventions and robust surveillance essential. The urgency of addressing malnutrition in all its manifestations is firmly established.

The simultaneous presence in a community of undernutrition alongside overnutrition, the so-called “double burden of malnutrition”, has escalated over recent decades, affecting people across age groups and income levels as societies undergo development and economic growth. This is a result of the “nutrition transition”, described as a shift in dietary habits away from varied and staple-based diets toward processed foods high in fats, sugars, and salt [2]. These dietary changes are frequently linked to globalization, rapid urban expansion, and more sedentary lifestyles. Unlike in the past, when such transitions unfolded gradually over centuries, recent decades have witnessed accelerated changes, with dietary diversity, nutritional disparities, and health risks increasing dramatically within a single generation. This shift has contributed to the “epidemiological transition”, whereby the public health burden moves from infectious diseases and undernutrition to a greater prevalence of overweight/obesity and NCDs. In parallel, the “demographic transition” involves populations becoming older on average, with elderly facing heightened risks of chronic diseases. As a result, policymakers confront unique challenges in simultaneously tackling undernutrition and overweight, along with their substantial health and economic ramifications [3].

The United Nations (UN) defined the 2030 Agenda, adopted in 2015 by the 193 Member States, to provide an overarching framework of 17 Sustainable Development Goals (SDGs) to steer global efforts toward social, economic, and environmental sustainability. At the heart of this agenda lies SDG 2-Zero Hunger which focuses on eradicating hunger, achieving food security and improved nutrition, ending malnutrition in all its forms, and fostering sustainable agricultural practices [4]. While certain advances have been made toward this goal, overall progress has been inconsistent and insufficient. Notable gains have occurred in densely populated nations with expanding economies; however, hunger, food insecurity, and malnutrition continue to rise in numerous regions worldwide. Many millions of people remain affected, especially in rural areas, where deep-seated poverty and chronic food insecurity persist [5]. Vulnerable groups—including women, young people, and Indigenous communities—experience disproportionate impacts from hunger, malnutrition, and associated health complications [6]. Despite global pledges, progress toward the broader goal of ensuring reliable access to sufficient and nutritious food for all has stagnated [7].

Accurately reporting the global number of people affected by malnutrition remains challenging due to variations in definitions, indicators, and measurement methods, as well as limitations in data quality and availability. Even within countries, coverage is uneven, with significant regional disparities and gaps at the subnational level. As a result, estimates are typically based on projections rather than precise measurements, and reported figures often differ across sources. For example, FAO’s prevalence of undernourishment relies on food balance sheets and household survey data, WHO uses child anthropometry to assess stunting and wasting, while the World Bank incorporates broader socioeconomic indicators. These methodological divergences can yield markedly different prevalence figures, complicating cross-country comparisons and sometimes leading to contested interpretations of trends. Despite these inconsistencies, it is unequivocal that hundreds of millions of people worldwide are affected by some form of malnutrition, whether it be undernutrition or overnutrition [8,9,10].

The ongoing lack of improvement in global food security and malnutrition rates casts doubt on achieving Zero Hunger by the 2030 deadline, a goal now just five years away. No country is immune: almost every country in the world is facing a serious nutrition-related challenge. Different forms of malnutrition can overlap and coexist. Currently, 88% of countries face a serious burden of either two or three forms of malnutrition [11]. Recognizing the gravity, scale, and complexity of the nutrition crisis is crucial for accurately assessing the current landscape and anticipating future trends, as well as for designing effective, evidence-informed, and sustainable policy responses that reinforce the urgent call to action.

### 1.1. Aim of the Study

This narrative review aims to provide a comprehensive and exhaustive overview of the topic, with a focus on current trends. It pursues several objectives. First, it analyzes and discusses some of the root causes underlying global food insecurity and malnutrition, synthesizing current evidence on prevalence and drivers. Second, it extensively evaluates progress toward the SDG 2 targets, identifying key gaps and persistent challenges. Finally, it highlights critical priorities for future research, policy, and investment in the remaining years leading up to 2030.

### 1.2. Review Approach

We conducted a narrative review of the literature published in the last 10 years. A search was performed on PubMed using the following query: “Food Insecurity” [MeSH] AND “Hunger” [MeSH], with filters applied for Books and Documents, Meta-Analyses, Reviews, Systematic Reviews. The search yielded 26 results. Titles and abstracts were screened to assess their relevance to the aim of the study. Following title and abstract screening, 4 articles were included in the review. In addition to the PubMed search, a manual search was carried out to identify further relevant articles (e.g., The Lancet series) and major global reports that were not retrieved through the electronic search. Our search was therefore expanded through a backward and forward literature search. This included also reports produced by United Nations bodies such as the Food and Agriculture Organization (FAO), the World Food Program (WFP), the United Nations Children’s Fund (UNICEF), and the UN Refugee Agency (UNHCR), as well as by international institutions such as the World Health Organization (WHO) and the World Bank.

## 2. Root Causes Underlying Global Malnutrition and Hunger

**Figure 1:**
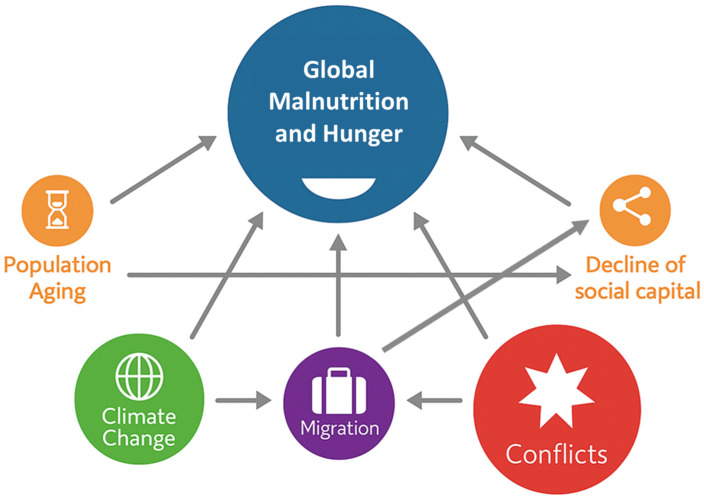
Major drivers of food insecurity and their interconnections in shaping and exacerbating global malnutrition and hunger.

The root causes of food insecurity and malnutrition are multiple, complex, and deeply interconnected. Figure 1 offers a visual representation of the interlinkages between the current major global crises, highlighting how these challenges often reinforce one another. The diagram features nodes of different sizes to reflect the relative impact or significance of each factor. The direction of the arrows explains the cause-effect interconnection. Some well-recognized drivers have not been explicitly represented in this figure. For instance, poverty and inequalities—although fundamental drivers of hunger and malnutrition—were not shown as separate elements, as they underpin and intersect with all the other drivers illustrated.

Climate change directly undermines food security and exacerbates existing vulnerabilities, such as migration pressures. Migration, in turn, places additional strain on social structures and contributes to a decline in social capital, a key component of community resilience to food insecurity [12].

The aged population is particularly vulnerable to malnutrition, not only due to physiological changes associated with aging, but also because of increased dependence on social and familial support. In the context of demographic transitions, where younger generations are insufficient to replace aging populations, social capital declines. This weakens the capacity of younger people to provide adequate economic and social support, thereby worsening the effects of food insecurity among the elderly.

Armed conflicts are another major drivers of food insecurity and undernutrition, often resulting from disruptions in food production, supply, and distribution. Conflicts cause fatalities and large-scale displacement, further eroding social capital and intensifying food insecurity.

The following chapters explore these key drivers of global malnutrition and hunger in greater depth.

### 2.1. Conflicts

Conflicts remain widespread around the world, although their nature has evolved in recent years, with a significant rise in civilian casualties. In 2023, the number of active conflicts reached 56—the highest recorded since World War II. That same year, civilian deaths from armed conflicts surged by 72%, exceeding 33,400 fatalities and marking the highest level since 2015 [13]. The causes and consequences of conflicts are complex and multifaceted, often stemming from a web of intertwined economic, environmental, political, cultural, and religious factors. The human, social, and economic costs of armed conflicts are profound and far-reaching.

Conflict remains the primary driver and magnifier of severe acute food insecurity. It directly disrupts food production, supply chains, and market access, while also weakening community resilience to additional shocks. Armed conflict can destroy or loot crops, livestock, and food stocks; interrupt trade and market activity, making it difficult for people to meet their basic nutritional needs. Moreover, occupation, repression, and the loss of human capital can indirectly impair food security by limiting access, availability, and utilization of food, driving up prices, and reducing economic investment [14]. Beyond immediate impacts, conflicts often reverse years of development gains, erode institutional capacity, and leave populations more vulnerable to future crises such as extreme weather events or economic downturns. These impacts can persist long after the fighting ends—especially in protracted conflicts—due to the destruction of infrastructure, loss of lives and livelihoods, environmental degradation, and disruption of essential services like education and healthcare.

The public health consequences of conflict further compound food insecurity. Evidence shows that countries affected by conflict bear a disproportionately high burden of infectious diseases such as polio and measles, due to damaged healthcare systems, interruptions in vaccine, and increased vaccine hesitancy [15]. These conditions create a vicious cycle between infectious diseases and malnutrition, placing the most vulnerable—including children, elderly, pregnant and lactating women, and refugees—at heightened risk of mortality. Malnutrition itself weakens immune function and is recognized as a major contributor to secondary immunodeficiency, sometimes referred to as nutritionally acquired immunodeficiency syndrome (NAIDS) [16].

Food is also frequently weaponized in conflict. It may be deliberately used to control or punish populations. A recent and tragic example is the situation in Gaza, where hundreds of Palestinians have been killed while attempting to access food aid [17]. At the same time, food insecurity can also act as a trigger or exacerbating factor for conflict. For instance, the protests that swept across North Africa and the Middle East in 2011 were partly fueled by rising food and energy prices.

Global conflicts also significantly undermine collective efforts to fight malnutrition. Resources are increasingly diverted from development and humanitarian programs to military spending, which reached a record high in 2024 [18]. Geopolitical tensions also hinder the international cooperation essential for addressing global challenges such as malnutrition. Moreover, mass displacements exert additional pressure on the resources of host countries, further complicating coordinated responses to food security.

According to the WFP, 10 of the world’s 13 worst food crises are currently driven by conflict. The situation is particularly dire in Gaza, where nearly 90% of the population—close to 2 million people—is experiencing food insecurity at crisis level or worse [12]. Overall, about 65% of people facing severe food insecurity live in fragile or conflict-affected regions, underscoring the inextricable link between armed conflict and hunger [19].

### 2.2. Climate Change

Climate change poses a significant threat to the progress achieved so far in reducing hunger and malnutrition. Nowadays, it stands as one of the most critical drivers of food insecurity in the 21st century, adversely affecting the availability, access, utilization, and stability of food systems over time. Rising temperatures, shifts in precipitation patterns, and the increasing frequency and intensity of extreme weather events—such as droughts, floods, and heatwaves—are diminishing agricultural yields and depleting global reserves of essential cereals. These impacts contribute to higher food prices, with agricultural communities among the most vulnerable to these changes [20].

The relationship between food security and climate change is deeply intertwined with equity considerations. Climate impacts differ across social groups depending on factors like age, ethnicity, gender, wealth, and social class. Extreme weather events have both immediate and lasting effects on the livelihoods of poor and vulnerable populations, heightening the risk of food insecurity and acting as a stress multiplier for internal—it could force 216 million people across six world regions to move within their countries by 2050 [21]—and cross-border migration [22].

The Intergovernmental Panel on Climate Change (IPCC) warns that a global temperature rise exceeding 1.5 °C could jeopardize up to one-third of global food production, with as much as 50% of agricultural output in tropical regions facing severe risks [23]. Climate change also introduces significant uncertainty about future water availability in many parts of the world. Additionally, shifts in pest and disease dynamics driven by warmer temperatures further threaten crop yields and the health of livestock. These climate impacts ripple through environmental, economic, and social systems, creating complex risks that extend beyond agricultural production alone [20]. As a result, rising food prices disproportionately burden the most vulnerable populations, particularly agricultural communities and smallholder farmers who often have limited capacity to adapt. Roughly 80% of the world’s population facing the highest risk of crop failures and hunger linked to climate change lives in Sub-Saharan Africa, South Asia, and Southeast Asia. In these regions, many farming households are already living in poverty and remain highly vulnerable. Severe droughts driven by El Niño events or broader climate shifts can push millions more into deeper poverty and food insecurity. If left unaddressed, climate change could drive as many as 130 million people into poverty by 2030, undoing decades of progress in development [21]. In Africa alone, it is estimated that around 43 million individuals could fall below the poverty line by 2030 due to these impacts [24].

### 2.3. Migration and Population Displacement

Migration has profoundly shaped the societies we live in today and remains an integral part of our shared history. Its causes and consequences are complex and multifaceted. While many individuals are compelled to leave their homes due to conflict, environmental shocks, or poverty, others migrate to pursue education, economic opportunities, family reunification, or employment to support relatives in their countries of origin. Migration plays a pivotal role in the structural transformation of economies, presenting both opportunities and challenges [25]. It occurs in various forms, with the majority of migrants relocating within their own countries. In fact, only about 1 in 30 people globally is an international migrant [26]. However, data on internal migration remain scarce, making it difficult to measure domestic movements accurately. A significant proportion of international migrants originates from rural areas or peri-urban communities.

Some migrants also cross international borders in search of work or a new life abroad. As of mid-2024, there were an estimated 304 million international migrants globally, representing 3.7% of the world’s population, up from 275 million in 2020. The top five countries of origin for international migrants were India (18.5 million), China (11.7 million), Mexico (11.6 million), Ukraine (9.8 million), and the Russian Federation (9.1 million). Notably, 53% of international migrants remained within their region of origin. Europe hosted the largest number of international migrants (94.1 million), followed by Asia (92.2 million), Northern America (61.2 million), Africa (29.2 million), and Latin America and the Caribbean (17.5 million). The top five destination countries for international migrants have remained unchanged since mid-2020: United States, Germany, Saudi Arabia, United Kingdom, and France [27].

Migration, whether voluntary or forced, has significant implications for global nutrition. Understanding the intricate relationships between migration, food security, agriculture, and rural development requires a nuanced analysis of the diverse forms that migration can take and the multiple factors driving it. The departure of the most dynamic members of communities may negatively affect productivity and local economies. However, international remittances, including financial or in-kind transfers sent by migrants to families and communities in origin countries, can help reduce poverty and improve household food security. In 2022, the top five recipients of remittances were India, Mexico, China, the Philippines, and Egypt [28].

Conversely, forced displacement often results in refugees living in camps or informal settlements, where they face heightened risks of severe food insecurity. Such displacement also indirectly impacts food security in host communities by placing additional pressure on local resources and services. Those migrants are among the most vulnerable populations, frequently exposed to heightened risks of malnutrition, ending up in areas with limited food availability or functioning markets, poor-quality diets, loss of jobs or livelihoods, and restricted access to healthcare [12].

Meanwhile, rapid urbanization, often driven by rural-to-urban migration, can affect agriculture production and strain urban food systems and increase exposure to unhealthy diets. With over 70% of the global population projected to live in cities by 2050, the impact of urbanization on food systems is profound [29]. This demographic shift influences dietary patterns in numerous ways, contributing to the nutrition transition marked by increased consumption of ultra-processed foods and rising rates of overweight and obesity.

### 2.4. Decline of Social Capital

The concept of social capital—understood as the norms and networks that enable individuals to act collectively—rose to prominence across the social sciences in the 1990s. Social capital encompasses the material and social benefits that emerge from relationships among individuals both within and between groups. Social capital is inherently multidimensional. One of the most comprehensive empirical studies by the World Bank identified six key dimensions: groups and networks, trust and solidarity, collective action and cooperation, information and communication, social cohesion and inclusion, and empowerment and political action. It also comprises cognitive, relational, and structural elements [30].

Evidence suggests that social capital can play a significant role in promoting food security by fostering cooperation, trust, and mutual support among community members throughout all stages of the food system—from production to consumption [31]. When social capital is lacking—often due to insufficient structural conditions or the erosion of community ties, such as through the outmigration of the workforce—communities face a higher risk of food insecurity. This impact is especially pronounced among vulnerable groups, including female-headed households, individuals living alone, adults with disabilities, and older populations. Social capital essentially represents the benefits that communities derive from interactions and relationships across diverse networks and groups. Higher levels of social capital are associated with greater resilience to shocks and crises, contributing to better food security outcomes [32].

In contexts that emphasize self-sufficiency and individualism—whether driven by urbanization, digital isolation, or political polarization—reliance on social support networks can carry social stigma or limitations, potentially reducing the effectiveness of social capital in addressing food insecurity [33]. Moreover, social capital in low-income countries evolves over time in response to economic, social, and environmental shifts. For instance, evidence indicates a decline in social capital in sub-Saharan Africa, driven by factors such as climate stress, changing livelihoods, and economic development. In this context, gender disparities can increase women’s vulnerability to food insecurity, creating a vicious cycle where social and economic disadvantages reinforce one another [34].

Drawing on this evidence, there have been calls for policies and interventions that strengthen social capital as a strategy to combat food insecurity and build more resilient communities.

### 2.5. Population Aging

Among older adults, physiological changes such as reduced appetite and diminished nutrient absorption significantly affect nutritional status. This population is particularly vulnerable to chronic diseases, which often increase nutritional needs while simultaneously reducing food intake and contributing to malnutrition. Malnutrition in older adults is linked to numerous comorbidities and functional impairments. Research highlights a bidirectional relationship between food insecurity and health outcomes in aging populations. Food insecurity increases the risk of developing chronic illnesses, while living with multiple chronic conditions, in turn, raises the likelihood of experiencing food insecurity [35]. This association may be partly due to the loss of income resulting from chronic disease and disability, which limits the ability to purchase adequate and nutritious food. Chronic health conditions can also restrict mobility and energy, making it more difficult for individuals to access food resources in the community or prepare healthy meals at home. Similarly, food insecurity has been identified as both a risk factor for and a consequence of frailty in older adults.

Beyond health concerns, older adults often face socio-economic challenges. Poverty and social isolation are significant contributors to malnutrition in this demographic. Factors such as migration and the erosion of social capital also play a crucial, though sometimes overlooked, role in food security [35]. Low levels of social capital can exacerbate poverty, social exclusion, and limited access to food assistance, ultimately reducing community resilience and the capacity to respond effectively to food crises [31].

In many countries—particularly high-income nations—the growing elderly population coincides with rising obesity rates, placing unprecedented strain on healthcare systems and increasing expenditures. Malnutrition in older adults imposes a significant burden on healthcare due to higher rates of complications and associated costs. According to the Global Burden of Disease Study 2021, by 2050 nearly a quarter of the global population living with obesity is projected to be over the age of 65 [36]. Older adults with obesity typically have greater healthcare needs due to the ongoing management of chronic conditions and specialized geriatric care. On the other hand, older adults affected by undernutrition are also more susceptible to increased healthcare utilization and costs, as a result of heightened risks of frailty and infections.

Demographic trends underscore the scale of this challenge. In 2050, the global population of people aged 60 and over is projected to be 2.1 billion, which is double the number in 2020 [37]. While this shift in distribution of a country’s population started in high-income countries, it is now low- and middle-income countries that are experiencing the greatest change. By 2050, two-thirds of the world’s population over 60 years will live in low- and middle-income countries [37].

As global populations age, new nutritional challenges emerge, and the number of older individuals at risk of food insecurity continues to grow.

## 3. The Status of Malnutrition and Food Insecurity in the World

Sustainable Development Goal 2 is about creating a world free of hunger, achieving food security and improved nutrition, and promoting sustainable agriculture by 2030. It consists of eight broad subgoals with specific targets on universal access to safe and nutritious food (2.1), end all forms of malnutrition (2.2), double the productivity and incomes of small-scale food producers (2.3), sustainable food production and resilient agricultural practices (2.4), maintain the genetic diversity in food production (2.5), invest in rural infrastructure, agricultural research, technology, and gene banks (2.6), prevent agricultural trade restrictions, market distortions, and export subsidies (2.7), and ensure stable food commodity markets and timely access to information (2.8) [38].

For the purpose of this review, we have analyzed specifically progress on targets 2.1 and 2.2.

### 3.1. Progress Toward SDG 2.1: By 2030, End Hunger and Ensure Access to All People

FAO is the main specialized agency of the United Nations focused on combating hunger, improving food security, and promoting sustainable agriculture worldwide through long-term development and policy work. FAO monitors the extent of global hunger using the uses the 3-year average prevalence of undernourishment, which is included in the 2030 Agenda as SDG indicator 2.1.1 to track progress towards achieving Zero Hunger [39].

When discussing food insecurity, we often refer to chronic hunger. Chronic food insecurity is defined as a long-term inability to consume sufficient food for a healthy and active life, usually driven by structural factors, including seasonal food shortages [40]. Addressing chronic food insecurity requires medium- and long-term improvements in both the quantity and quality of food consumption. Despite global efforts, the world remains off track to meet this goal.

**Figure 2:**
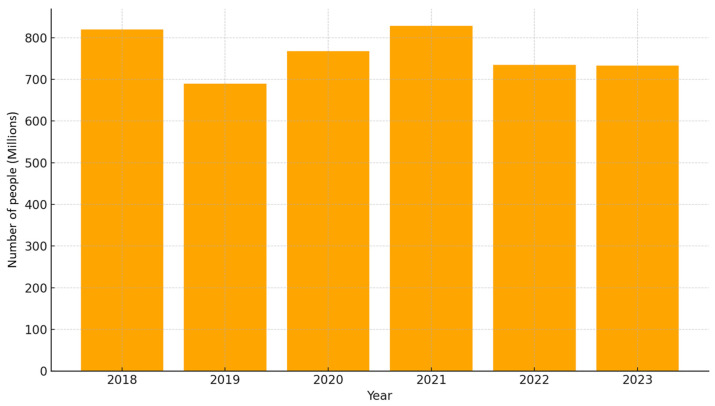
Number of undernourished people facing food insecurity over a six-years period (2018–2023).

Figure 2 illustrates global hunger trends over the past six years, with data available up to 2023 according to The State of Food Security and Nutrition in the World (SOFI) [7].

Food security is dynamic. Although precise numbers are difficult to determine due to data gaps and varying definitions, it is estimated that the number of undernourished people worldwide increased sharply between 2019 and 2021, partly due to the impacts of the COVID-19 pandemic. In 2022, a slight improvement was observed, with the number of undernourished people estimated at 735 million. However, this figure has remained largely unchanged for three consecutive years, with the latest estimates for 2023 indicating between 713 million and 757 million people facing hunger globally—representing 9.2% of the global population and equating to roughly one in eleven individuals—with the heaviest toll borne by populations in low- and middle-income countries (LMICs), such as in Africa (one out of every five people) and regions grappling with conflict and political instability [9]. This level is far from the SDG 2.1 target of zero hunger and underscores the severity of the current food security crisis [7].

Food insecurity continues to disproportionately affect populations in many regions of the world, particularly in rural areas and among vulnerable groups such as women, youth, the elderly, and Indigenous peoples. Africa has the highest share of its population facing hunger, with 20.4% affected, and the prevalence continues to rise. Meanwhile, Asia remains home to the largest absolute number of undernourished people—around 384.5 million—a figure that has remained relatively stable, accounting for more than half of the global total. While the number of undernourished people is increasing in Africa, some progress has been made in Latin America and the Caribbean. Nevertheless, in all regions, hunger levels remain above those recorded before the COVID-19 pandemic. If current trends persist, projections suggest that by 2030 approximately 582 million people could still be chronically undernourished, with over half residing in Africa [41].

Additionally, some countries or areas—particularly those experiencing shocks, conflicts and/or instabilities—are identified as “Hunger Hotspots” where acute food insecurity is a major concern and requires external assistance for food [42]. Acute hunger, defined as severe food deprivation occurring at a specific point in time and posing immediate threats to life or livelihoods, regardless of the cause, context, or duration. The classification of acute food insecurity focuses on identifying areas with substantial food consumption gaps requiring urgent action to save lives and protect livelihoods. This is typically measured as Integrated Food Security Phase Classification (IPC) Phase 3 (Crisis) or higher. Such situations demand immediate interventions to reduce food gaps, safeguard livelihoods, and scale up the treatment and prevention of acute malnutrition among affected populations [40]. Specifically, “FAO in emergencies” describes the organization’s dedicated work and operational efforts during humanitarian crises. It focuses on responding rapidly and strengthening resilience when conflicts, natural disasters, disease outbreaks, or climate shocks threaten food security and agriculture. Its activities are often coordinated as part of broader humanitarian responses alongside partners such as WFP, UNICEF, and UNHCR [43].

**Figure 3:**
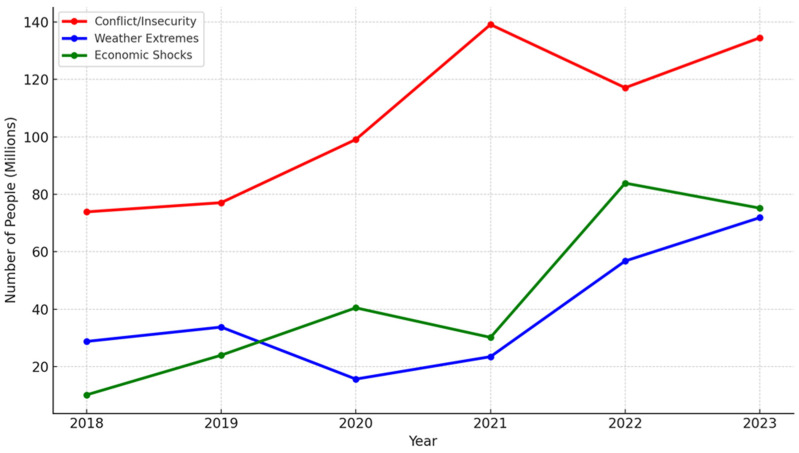
Trends in the number of people experiencing Food Crisis (IPC phase 3 or above) over a six-years period by predominant driver. (Source: revised from FSIN, GRFC 2019–2023) [12].

The number of people facing high levels of acute food insecurity—and thus requiring urgent food and livelihood assistance—has increased each year, rising from 193 million in 2021 [44] to nearly 295.3 million in 2024 across 53 countries and territories analyzed. This represents a nearly 5% increase from 2023. Over the years, the prevalence of acute food insecurity has almost doubled, rising from 14% in 2018 to a peak of 22.6% in 2024. This reflects worsening conflict-driven crises and the intensifying effects of weather extremes and economic shocks. Despite acute food crises arises from a combination of interconnected and mutually reinforcing drivers, rather than from a single shock or hazard, the Global Report on Food Crises [12] identifies a predominant driver for each country or territory. As illustrated in Figure 3, the report presents the number of people in IPC Phase 3 or above over the past six years, disaggregated by main driver (conflict, weather extremes, or economic shocks).

As shown in the graph, conflict has remained the most significant driver of acute food insecurity, exhibiting an overall upward trend in recent years. The only notable decline occurred between 2021 and 2022, primarily due to the impact of the COVID-19 pandemic, which led to a sharp increase in economic shocks and temporarily shifted the predominant driver of acute food insecurity. In 2023, conflict once again became the leading driver of food crises in 20 countries and territories, affecting 134.5 million people. Weather extremes were the primary driver in 18 countries, where 71.9 million people were affected. The trend for weather-related shocks is particularly striking, underscoring the rapidly growing impact of climate change and the increasing number of people affected in recent years. Moreover, economic shocks (including the indirect impact of COVID-19 until 2021 and the effects of the war in Ukraine in 2022) were a significant driver in 21 countries, impacting 75.2 million people [12].

As for 2025, the WFP estimates that 319 million people will face acute food insecurity, including 120 million in Eastern and Southern Africa, 68 million in Asia and the Pacific, 58 million in Western and Central Africa, 38 million in the Middle East, Northern Africa, and Eastern Europe, and 34 million in Latin America and the Caribbean [45]. Acute food insecurity in particular demands urgent interventions in countries such as Gaza and Sudan, as well as in pockets of South Sudan, Haiti, and Mali.

### 3.2. Progress Toward SDG 2.2: By 2030, End All Forms of Malnutrition

Malnutrition, in all its diverse forms, stands as the primary driver of poor health worldwide. Across every stage of life, malnutrition poses a significant threat, leaving deep impacts on individual health, development trajectories, and economic progress. Progress towards the SDG2.2 targets can be monitored through the voluntary global nutrition goals endorsed by WHO member states [11].

**Figure 4:**
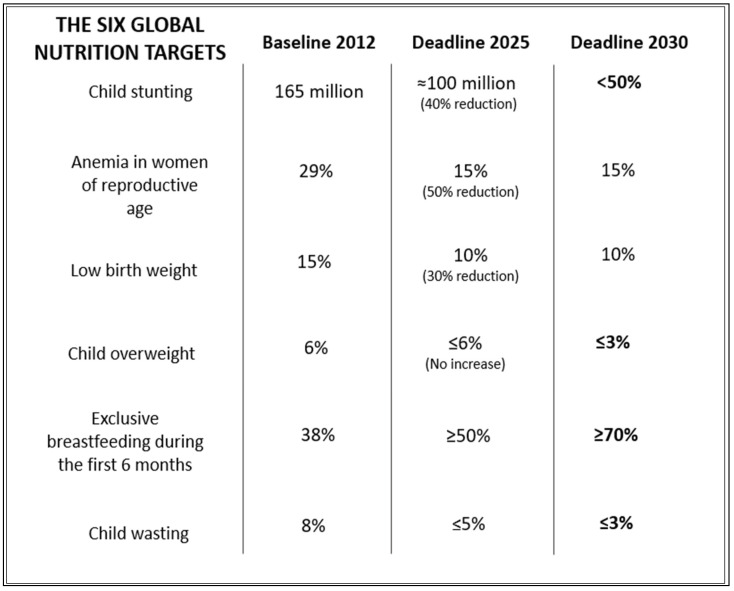
The six global nutrition targets, from baseline to deadlines.

Specifically, in 2012, the World Health Assembly established six Global Nutrition Targets (GNTs)—covering child stunting, child wasting, anemia among women of reproductive age, low birth weight, childhood overweight, and exclusive breastfeeding—to serve as critical indicators reflecting maternal and child health [46]. These targets were chosen due to their epidemiological significance, public health implications, and the existence of proven and practical public health interventions. The GNTs were designed to spur governments and policymakers into action to address interconnected nutrition issues, with an initial deadline set for 2025. By 2017, WHO advocated extending these targets to 2030, proposing some modifications. Specifically, the revised goals aimed to halve the number of stunted children, reduce wasting prevalence to below 3%, lower global childhood overweight rates to under 3%, and raise exclusive breastfeeding rates to at least 70% (Figure 4) [47]. Achieving the GNTs by 2030 is central to the United Nations’ SDG of eradicating hunger by that year.

Furthermore, in 2013, the World Health Assembly acknowledged that people’s living and working conditions, along with their lifestyle choices, significantly shape health outcomes and quality of life. Consequently, it formulated the Global Action Plan for 2025, addressing nine diet-related targets for non-communicable diseases (NCDs) [48]. This plan focuses on four main types of NCDs—cardiovascular diseases, cancer, chronic respiratory diseases, and diabetes—which contribute the most to the global NCD burden in terms of illness and mortality. Additionally, it targets four shared behavioral risk factors: tobacco use, unhealthy diets including excessive salt/sodium intake, physical inactivity, and harmful alcohol consumption [49]. In 2019, the 72nd World Health Assembly prolonged the NCD Global Action Plan through 2030 to align it with the broader 2030 Agenda, aiming to support also progress towards SDG 3.4 (reduce by one third premature mortality from non-communicable diseases through prevention and treatment and promote mental health and well-being) [50].

### 3.3. A Global Overview

The Global Nutrition Report—the world’s leading independent assessment of nutrition progress—highlights that advances remain insufficient, with unacceptably high levels of malnutrition persisting worldwide. According to the report, the world is not on track to achieve any of the six Global Nutrition Targets [49].

The prevalence of stunting among children under five has declined steadily, falling from 26.3% (equating to 177.9 million children) in 2012 to 22.3% (148.1 million children) in 2022 [49]. However, the most recent data indicate a slight reversal, with the number rising to 149.2 million in 2023 [10]. Nevertheless, current progress is insufficient for achieving the 2030 target of halving the number of stunted children under five. If existing trends continue, projections indicate that 19.5% of children under five will be stunted in 2030, exceeding the global target of 13.5% [7]. A slower reduction means that significant numbers of children and adolescents will continue to endure lifelong consequences associated with early childhood stunting.

According to the same report, globally, the prevalence of wasting among children under five has shown little change over the past ten years. In 2012, 7.5% (50.7 million children) were wasted, decreasing slightly to 6.8% (45 million children) by 2022. Based on current progress, the world is not on track to meet the 2030 global target of reducing wasting prevalence to 3%, as projections suggest that 6.2% of children under five will be affected in 2030—more than double the target level.

The prevalence of overweight among children under five has largely stagnated. In 2012, it was 5.5% (37 million children) and remained virtually unchanged at 5.6% (37 million) in 2022 [49], with the most recent data reporting 38.9 million overweight children [10]. Projections for 2030 suggest that 5.7% of children under five will be overweight, nearly double the global target of 3%. Those children are at increased risks of developing obesity and NCDs in later life. At present, more than 40% of adults worldwide—approximately 2.2 billion men and women—are classified as overweight or obese [10].

Little to no progress has been made in reducing low birth weight among newborns, with prevalence shifting only slightly from 15% (21.6 million) in 2012 to 14.7% (19.8 million) in 2020—the most recent year for which data exists [49]. Trends suggest that by 2030, 14.2% of newborns globally will have low birth weight, falling short of the global goal of a 30% reduction from baseline levels, equivalent to achieving a prevalence of 10.5% by 2030 [7].

Substantial improvement has been recorded in exclusive breastfeeding rates for infants under six months old. Recent estimates indicate an increase from 37.1% (25.7 million infants) in 2012 to 48% (31.3 million) in 2022. However, despite this progress, the world remains off track to reach the 2030 target of 70% exclusive breastfeeding, with current projections indicating a prevalence of only 59% by that time [7].

Globally, anemia among women aged 15 to 49 rose from 28.5% (520 million women) in 2012 to 29.9% (571 million) in 2019, reaching 32.3% in 2022, according to a study by the Global Nutrition Target Collaborators. Projections suggest anemia prevalence will remain at 32.3% in 2030, failing to achieve the target of a 50% reduction [7].

Progress is also lacking across all nine diet-related NCD targets [51]. Childhood obesity closely correlates with adult obesity [52]. Over the past 30 years, obesity prevalence among children and adolescents has surged by 244%, with forecasts indicating an additional increase of 121% over the next three decades [53]. As a result, adult obesity prevalence trends are unlikely to be reversed; actually, 2.2 billion people globally are overweight (12.3%), of whom 772 million are affected by obesity [49]. Without swift, effective measures, overweight and obesity are projected to continue increasing worldwide, with estimates placing adult obesity prevalence at 19.8% in 2030 [7].

Additionally, 538.7 million people have diabetes (10.5% men and 8.9% women), and 1.2 billion people have raised blood pressure (24.0% men and 19.9% women). The world is also off track to meet the target of a 30% relative reduction in mean population intake of salt/sodium, with the trend rising. The most recent available data indicate that adults consume an average of 2.89 g of sodium daily (expected to increase by years), whereas the target is 2.01 g [49].

### 3.4. Regional Overview

Across the global nutrition targets, more countries are currently off track than on track. At present, according to data from the Global Nutrition Target Collaborators, three-quarters of countries worldwide (146 out of 195) are not on track to meet the target for reducing low birth weight. This figure excludes nations with insufficient data for assessing progress, which may also be off track. 72.8% of the world’s newborns reside in countries failing to meet the low-birth-weight goal [54]. Projections suggest that, by 2030, no country will meet the target [54], with 14.2% of newborns expected to have low birth weight, missing the goal of reducing prevalence to 10.5% by 2030 [7].

More than 40% of countries (82 out of 195) are off track for reaching the exclusive breastfeeding target, and in 88 countries, insufficient data prevent progress assessments. Over half of infants under six months old (54.2%) live in countries currently off track. Prevalence estimates and projections to 2030 have not been developed for high-income countries, where concerns persist about low exclusive breastfeeding prevalence [54].

Half of the world’s countries (96 out of 195) are off track in pursuing the stunting target, with 75.1% of children under five living in these countries. For 40 countries, data are insufficient to assess progress toward the stunting goal.

More than a quarter of countries (55 out of 195) are off track for achieving the wasting target, with over half of children under five (54.7%) residing in those nations. Seventy-two countries, accounting for 7.3% of the global population, lack sufficient data for monitoring progress on wasting.

Approximately 60% of countries (119 out of 195) are off track for the childhood overweight target. For another 37 countries, data are insufficient for assessing progress. Countries off track for the overweight target account for half of the global population of children under five (52.5%).

Nearly all countries globally (191 out of 195) remain off track to achieve the target for reducing anemia among women.

As for diet-related NCD targets, only a few high-income Western nations are projected to achieve targets for raised blood pressure and diabetes, while in the African region, no country is currently on track to meet any of the diet-related NCD targets [49]. Specifically, almost every country (191 out of 195) is off course in addressing global adult obesity targets. China currently has the largest absolute number of adults living with overweight and obesity, followed by India and the United States. In Asia and Africa, rising populations are contributing to a substantial increase in individuals affected by overweight and obesity. If past trends persist, the GBD 2021 Adult BMI Study forecasted that the total number of adults living with overweight and obesity by 2050 will reach nearly 4 billion, over half of the likely global adult population at that time. While China, India, and the USA will continue to account for a significant share of this burden, the sub-Saharan Africa super-region is projected to experience a 254.8% increase in the number of adults living with overweight and obesity [54]. Furthermore, no country is on track to achieve the targets for reducing salt intake or halting the growth of adult obesity rates. As a result, the burden of various NCDs—including diabetes, cardiovascular diseases, and cancers—is expected to continue increasing.

## 4. Nutrition at the Heart of All SDGs

There is a significant opportunity to advance the SDGs by fostering stronger connections across traditionally separate sectors. Some relationships between specific targets can be constraining or even counteractive, hindering progress toward other goals. Food security is a fundamental component of sustainable development and is deeply interconnected with all other SDGs.

**Figure 5:**
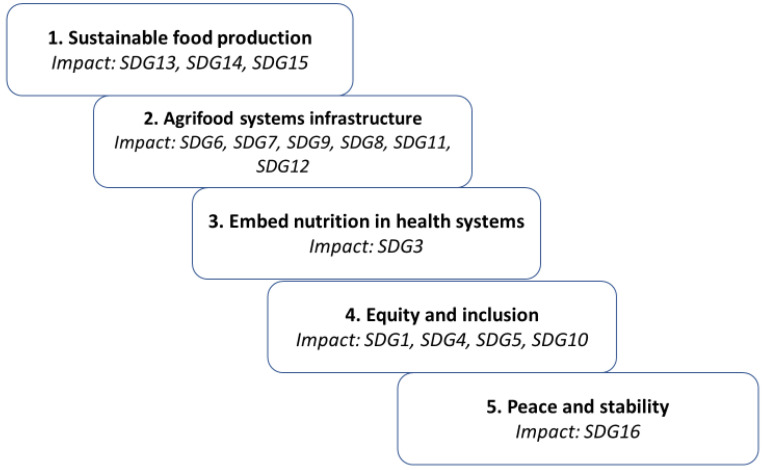
The five key domains critical for achieving better nutrition.

The SDGs can be grouped into five key domains critical for achieving better nutrition [11] (Figure 5):

- Sustainable food production is essential for maintaining resilient land and water systems capable of supporting the diversity required for nutritious and healthy diets. Adopting more sustainable dietary patterns could substantially influence climate change (SDG13) and help preserve biodiversity (SDG14 and SDG15).
- Systems infrastructure is crucial, involving sustainable solutions for agriculture and food production (SDG12), access to clean water (SDG6), clean energy (SDG7), and affordable technology (SDG9). At the same time, economic transformation can contribute to greater nutrition security and sustainable agriculture (SDG8), with benefits extending to urban, peri-urban, and rural communities (SDG11).
- Health systems play a vital role in delivering treatment and preventive measures that improve nutrition on a large scale. Better nutrition helps reduce illness and mortality rates, thereby alleviating pressure on health systems (SDG3).
- Equity and inclusion are indispensable to ensure that efforts to reduce main causes of food insecurity such as poverty (SDG1), reach gender inequalities (SDG5) and address inequalities between and among communities (SDG10), expand education (SDG4).
- Peace and stability are fundamental for securing food access. The proportion of undernourished people living in conflict-affected or crisis-prone regions is significantly higher than in other areas. Without peace and stability, eliminating malnutrition remains unattainable (SDG16).

Understanding these interconnections and implementing targeted strategies are essential steps toward achieving Zero Hunger and ensuring food security for all. Nutrition is both a prerequisite for achieving several SDGs and a goal supported by progress in other SDGs. Malnutrition and food insecurity create obstacles to achieving specific SDGs, and conversely, improvements in those goals help enhance nutrition outcomes [55]. For instance, poverty significantly increases vulnerability to malnutrition, while malnutrition itself perpetuates poverty (SDG1). Adequate nutrition lowers the risk of disease, reducing morbidity and mortality (SDG3), and supports cognitive development and educational attainment (SDG4). Nutrition also contributes to gender equality (SDG5). The link between nutrition and gender is profound: of the more than 300 million people experiencing extreme hunger globally, nearly 60% are women and girls. In many LMICs, women often consume less diverse and lower quantities of nutritious food compared to men, despite requiring greater nutritional support during specific life stages such as pregnancy and breastfeeding. According to a recent UN statement, achieving full gender equality could take nearly 300 years if progress continues at its current pace [56]. More broadly, nutrition helps reduce inequalities within and among countries (SDG10), particularly for vulnerable populations, smallholder farmers, and marginalized groups. Ensuring healthy diets for all could also boost productivity by creating healthier workforces (SDG8).

Food production and consumption have major implications for environmental sustainability. Sustainable food production is key to nutrition outcomes. Clean water and sanitation (SDG6), together with robust infrastructure systems (SDG11), are essential to secure safe, nutritious, and healthy diets. Food production is a significant source of global greenhouse gas emissions. Agricultural yields will decrease as temperatures increase by more than 3 °C. Increased carbon dioxide will result in decreased protein, iron, zinc, and other micronutrients in major crops consumed by much of the world [11]. Reducing dependence on fossil fuels can lower these emissions, decrease environmental pollution, and promote food security (SDG7). Diets and food systems also influence consumption and waste patterns (SDG12), contribute to climate change (SDG13), and affect marine (SDG14)—unsustainable fishing threatens 17% of the world’s protein and a source of essential micronutrients—and terrestrial ecosystems (SDG15). Conversely, affordable access to technology and infrastructure is crucial for agricultural development and food security (SDG9). Peace and justice are vital to ending food insecurity and malnutrition (SDG16). Strengthening collaboration, partnerships, and financing mechanisms is critical for eradicating malnutrition in all its forms (SDG17). This effort requires engagement from diverse actors, including policymakers, private-sector stakeholders, and individual consumers [11].

## 5. Discussion

Recognizing the urgency of achieving the full set of Agenda 2030 targets is crucial as the deadline approaches. However, Sustainable Development Goal 2, which encompasses food security and nutrition, continues to face major obstacles. Despite notable progress toward global nutrition targets, current efforts remain insufficient, and only a limited number of countries are on track to meet these objectives by 2030. Projections indicate that by 2030, 21 countries will meet two of the six global nutrition targets, 94 countries will meet only one, and 89 countries are expected to meet none. Looking ahead to 2050, it is estimated that 77 countries will achieve two or more targets, 94 countries will remain on track for only one, and 33 countries—mainly located in central and western sub-Saharan Africa—will not meet any targets at all [54]. Progress has been uneven across regions and population groups, exposing persistent disparities and deep-rooted structural barriers. It should also be considered that the absence of a unified global source of data on food insecurity and malnutrition, combined with cross-country differences, hinders a precise assessment of the problem’s magnitude. This increases the risk of underestimating its true impact and delaying appropriate policies and interventions. Moreover, reported statistics are not neutral; they are shaped by political and socio-economic contexts, including the capacity of national statistical systems, donor priorities, and international accountability frameworks.

**Figure 6:**
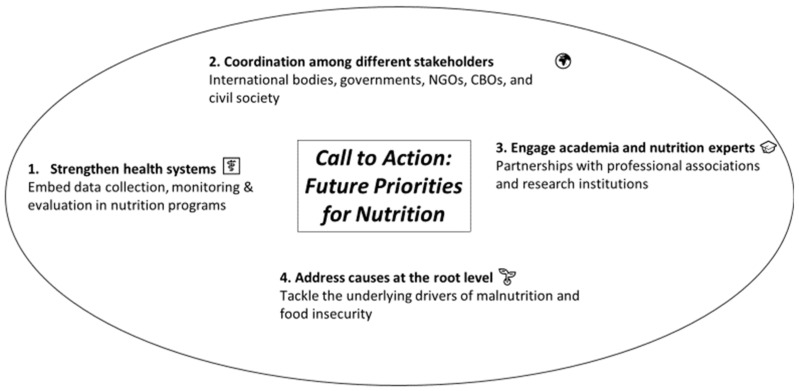
Key priorities to accelerate the Global Nutrition Targets.

Achieving SDG 2-Zero Hunger will require significantly scaled-up, better-targeted, and cost-effective investments, alongside stronger political commitment, scientific innovation, and international cooperation (Figure 6). To support these actions, global and national nutrition stakeholders have common priorities to tackle the nutrition agenda [57,58].

An integrated approach is urgently needed, one that acknowledges the intrinsic interconnections between food systems and sustainable development. The multifaceted nature of food security and nutrition demands a shift in the financial landscape, moving beyond fragmented and siloed approaches. Financial actors should embed food and nutrition priorities into broader development financing and investment frameworks.

To effectively combat undernutrition at scale, an additional $13 billion annually will be required over the next decade (2025–2034) [59]. This investment is projected to generate $2.4 trillion in economic benefits, highlighting the direct link between nutrition and economic growth. In fact, nutrition is foundational infrastructure—critical for brain development, human productivity, and long-term prosperity [11]. For every dollar invested in this infrastructure—tackling undernutrition and thereby supporting human development throughout life and enhancing mental and productive capacity—there is an expected return of $23. These economic gains far outweigh the estimated $41 trillion in costs that could result from inaction over the same 10-year period [59].

However, recent developments raise significant concerns. The announcement that over 80% of United States Agency for International Development’s (USAID) nutrition programs will be terminated threatens both immediate and long-term gains in global health and development, particularly in low- and middle-income countries. Many of these programs—especially those targeting child malnutrition—have proven highly effective in reducing child mortality [60]. In the wake of the U.S.’s decision, several other Western donor countries have announced similar aid reductions over the next 3–5 years, posing a serious threat to decades of progress [61]. The consequences are already tangible: the WFP has closed its Southern Africa office, leaving 27 million people at increased risk of hunger amid the region’s worst drought in decades [62]. Without adequate time and resources to implement adaptive strategies, the most severe impacts cannot be mitigated and risk severely undermining the achievement of the 2030 Agenda.

In moving forward, actionable recommendations should be explicitly tailored to the responsibilities of different stakeholders at international, national, and non-governmental levels. For policymakers, it is crucial to integrate nutrition services effectively into national health systems and universal health coverage, balancing global guidelines with what is appropriate and feasible at the local level. Government authorities should also actively engage civil society, enabling organizations to demand change and, above all, to serve as a catalyst in formulating and implementing innovative nutrition policies. Civil society participation is equally important for promoting social mobilization around nutrition. Empowering civil society and community-based organizations (CBOs) should not be regarded as a temporary measure but as an investment in lasting capacity, ensuring a continuous voice in decision-making processes. By working closely with government authorities, civil society can impart sustainability to interventions, help projects remain aligned with national priorities and guidelines, and strengthen interaction between communities and district management. Such collaboration allows for the exchange of views on real conditions at the grassroots level and supports the implementation of protocols within communities.

The scientific and research community also has a pivotal role in producing context-specific evidence on effective interventions, developing innovative delivery platforms, and advancing research on the intersections between nutrition, climate change, and conflict. These insights are indispensable for guiding adaptive strategies and informing policy decisions [63]. Academia, in particular, can help bridge the gap between theory and practice by translating guidelines and research evidence into field applications. As an incubator of knowledge and innovation, academic institutions have been directly called upon to contribute to achieving the United Nations Sustainable Development Goal of Zero Hunger (SDG 2) by 2030 [64]. A relevant example is UNICEF’s conceptual framework of the “Triple-A” cycle, which guides decision-making to improve the effectiveness of nutrition programs: a program must first be Assessed, its causes and problems Analyzed, and then Actions to be taken to address these problems based on the analysis and available resources, with the cycle repeated to evaluate implementation effectiveness [65]. Academia can play a central role in operationalizing this approach and applying it to existing or future nutrition programs at the local level, implemented either by governmental or non-governmental organizations. Interactive dialogue between research and social mobilization can create a mutually reinforcing process, enriching the scientific field while strengthening social action [66]. For example, the current body of evidence on climate change and food insecurity remains limited, with insufficient geographical and economic diversity, restricted generalizability, and a lack of granular understanding of underlying mechanisms and feasible adaptation strategies. Further research is needed to clarify these mechanisms and to identify economically viable solutions across diverse geographical and sociocultural contexts, as well as in response to a variety of climate-related shocks [67].

In parallel, strengthening international cooperation and pursuing peaceful diplomacy are essential to eliminating the underlying drivers of malnutrition and food insecurity—poverty, inequality, conflict—while also enhancing humanitarian response capacity in the face of acute crises [68]. For international donors and development partners, safeguarding and expanding funding for nutrition must be considered a long-term commitment rather than a discretionary expense. Integrating nutrition priorities within wider development financing frameworks, coupled with continued strong support for humanitarian agencies and non-governmental organizations, is essential to safeguarding progress, especially in fragile and conflict-affected settings [69,70]. Furthermore, while many drivers of food insecurity and health inequalities (i.e., the social determinants of health) lie outside the health sector, it is well recognized that national health systems—and primary care in particular—have a crucial role to play in mitigating these inequalities [71].

### Limitations

This study has several limitations that should be acknowledged. As a narrative review, it does not follow a systematic and reproducible methodology, which may introduce selection bias. Some relevant studies may have been missed, particularly those published in languages other than English or not indexed in PubMed. Second, while the inclusion of official reports from international agencies ensured broader coverage, it also introduced heterogeneity in definitions, indicators, and measurement methods, complicating direct comparisons across sources and limiting the ability to draw consistent conclusions. This restricts the generalizability of the findings, especially in fragile and conflict-affected settings where data collection is scarce or inconsistent. Finally, the dynamic nature of the topic means that projections remain highly uncertain and may change rapidly in response to global crises. Despite these limitations, this review synthesizes key evidence and highlights critical research and policy gaps that must be addressed to accelerate progress toward the 2030 Agenda. Failure to act decisively will result not only in increased rates of morbidity and mortality, but also in long-term damage to human capital and societal development. These consequences will reverberate across generations, disproportionately affecting the world’s most vulnerable populations.

## 6. Conclusions

The path to achieving Zero Hunger is a shared global responsibility that demands the active engagement of governments, international organizations, civil society, and the private sector. Equally vital are strong partnerships between research institutions, universities, nutrition associations, national governments, non-governmental organizations, community-based organizations and civil society to drive the development and implementation of evidence-based policies and programs. Meaningful progress toward ending hunger by 2030 can only be achieved through coordinated, multisectoral, evidence-informed, and peace-oriented interventions.

Moreover, achieving SDG 2-Zero Hunger is not only a goal in itself, but also a cornerstone for progress across the entire Sustainable Development Agenda, given its foundational role in health, education, equity, and economic development. Our collective future depends on these efforts. Only by working together can we ensure that no one is left behind.

## Data Availability

No new data were created or analyzed in this study. Data sharing is not applicable to this article.

## Abbreviations

The following abbreviations are used in this manuscript:

CBOs	Community-based Organizations
FAO	Food and Agriculture Organization
GBD	Global Burden of Disease
GNTs	Global Nutrition Targets
IPC	Integrated Food Security Phase Classification
IPCC	Intergovernmental Panel on Climate Change
LMICs	Low- and middle-income countries
NAIDS	Nutritionally acquired immunodeficiency syndrome
NGOs	Non-governmental organizations
NCDs	Non-communicable diseases
SDGs	Sustainable Development Goals
SOFI	State of Food Security and Nutrition in the World
UN	United Nations
UNHCR	UN Refugee Agency
UNICEF	United Nations Children’s Fund
USAID	United States Agency for International Development
WFP	World Food Program
WHO	World Health Organization
